# Acute esophageal necrosis syndrome as a rare complication of diabetic ketoacidosis

**DOI:** 10.2478/jtim-2023-0144

**Published:** 2024-05-21

**Authors:** Khalil El Ali, Lotfi Triki, Sébastien Redant, Joe Kadou, Rachid Attou

**Affiliations:** Intensive Care Department, Centre Hospitalier Universitaire Brugmann, Brussels, Belgium; Emergency Department, Centre Hospitalier Interrégional Edith Cavell, Brussels, Belgium

## To the editor

**Figure 1:**
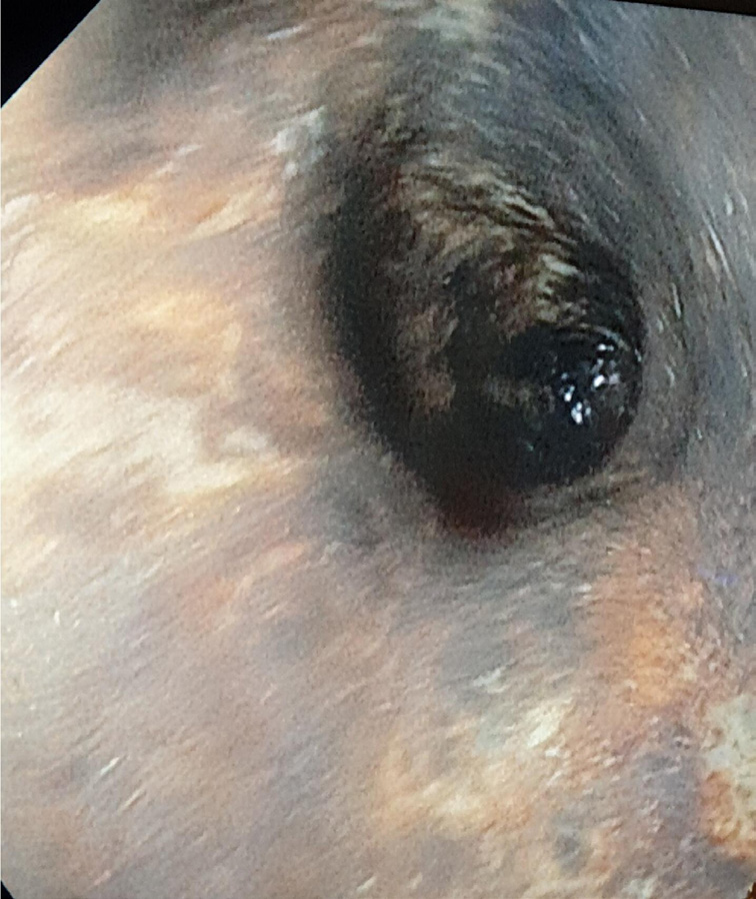
“Black eosophagus”, extensive necrotic lesions along the entire length of the esophagus.

A 78-year-old patient is admitted for hematemesis. He describes brownish vomiting, associated with blood clots, epigastric and retrosternal pain. This patient is known for non-insulin-requiring diabetes treated with metformin and gliquidone. He had mottling of the knees. The abdomen is soft, depressed, slightly painful in the epigastric area without defensiveness or rebound. Peristalsis is present. Arterial blood gas revealed metabolic acidosis with pH 7.24, pCO_2_ 25 mmHg, pO_2_ 106 mmHg, blood glucose 553 mg/dL and lactate 3.5 mmol/L. We also note an acute renal failure, with urea at 226 mg/dL, creatinine at 5.3 mg/dL and a creatinine clearance estimated at 11 mL/min/1.73 m^2^. Hepatic function was normal with INR at 1.09, albumin 38 g/L, ALT 26 UI/L, AST 32 UI/L and bilirubin 1.6. Urine analysis showed the presence of ketone. The patient received an infusion and intravenous insulin therapy started at 5 UI/h. Cardiac ultrasound showed a preserved left ventricular ejection fraction, a left ventricular kissing with low filling pressures. A gastro-intestinal endoscopy revealed a black esophagus along its entire length (Figure 1), as well as ischemic necrosis without signs of active bleeding. The diagnostic hypothesis at this stage is an ischemic necrosis of the esophagus (“black esophagus” or Gurvitis syndrome), due to a probable low flow following severe hypovolemia in a context of diabetic acid-ketotic decompensation.

During the hospitalization, the evolution was marked by major swallowing disorders requiring a gastrostomy. Vasculitis and thrombophilia tests were negative. An angiovascular CT thoracic didn’t reveal any vascular obstruction. He was discharged from the hospital after a stay of about one month.

Acute esophageal necrosis or “black esophagus” was first described in 1990 by Golberg *et al*.^[1]^ It is characterized by a black appearance of the esophageal mucosa extending from the gastroesophageal junction and may reach the entire length of the esophagus. The lower third of the esophagus is affected in 97% of cases, reflecting a less abundant vascularization. ^[2]^ The incidence is estimated between 0.01% and 0.28%.^[3,4]^ It affects men preferentially with a peak of incidence in the sixth decade. Risk factors are immunocompromised patients, diabetic, neoplasia, hypertension, coronary artery disease, antiphospholipid syndrome^[3,4]^ and alcohol abuse.^[2]^ The symptoms are upper GI bleeding with hematemesis and melena, dysphagia, abdominal pain, hemodynamic instability. Differential diagnoses include malignant melanoma, melonocytosis, pseudomelanosis, acanthosis nigricans, and caustic ingestion. The major complications are esophageal perforations (7% of cases) and esophageal strictures (25%–40% of cases). Mortality is around 6%.

In our case, the patient presents a state of severe hypovolemia on admission. The combination of hypovolemic state and gastric reflux caused by vomiting, explains the etiology of acute esophageal necrosis. Moreover, diabetes predisposes to the formation of atherosclerotic plaque and the development of neuropathic gastroparesis. Care giver must pay attention to the hypovolemia responsible of hypotension, shock but also organ dysfunction and necrosis.

**Figure 2:**
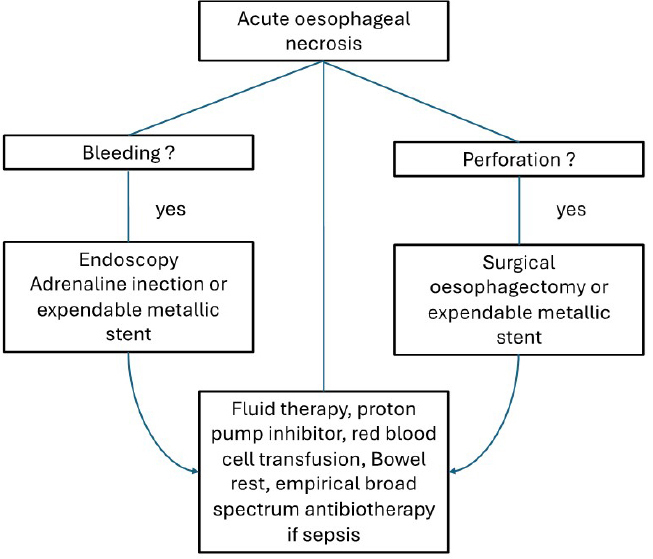
Simplified algorithm for management of acute esophageal necrosis and its complication.

Dias *et al*.^[5]^ proposed an algorithm for management (Figure 2) including aggressive rehydration, proton pump inhibitors, parenteral nutrition, empirical broad-spectrum antibiotic therapy. A follow-up endoscopy should be performed four weeks after the initial event to exclude late complications. The use of nasogastric tubes should be avoided because of the increased risk of esophageal perforation.

## Conclusion

Acute esophageal necrosis or “black esophagus” is a rare pathology whose pathophysiology requires a combination of events leading to ischemia of the esophageal mucosa. This condition is frequently related to a hyperglycemic state.
